# Australian medical student expectations of work-life balance as a doctor

**DOI:** 10.15694/mep.2020.000256.1

**Published:** 2020-11-17

**Authors:** Sarika Suresh, Rebekah Hoffman, Sue Liu, Andrew Gosbell

**Affiliations:** 1 Concord Hospital; 2 University of Wollongong; 3 Monash University; 4 General Practice Registrars Australia

**Keywords:** Medical student, wellbeing, work-life balance, burnout

## Abstract

This article was migrated. The article was marked as recommended.

**Objectives:** To explore the perceptions of medical students on achieving good work-life balance after graduation, and their opinions on parenting having an impact on their future careers.

**Methods:** Cross-sectional cohort study of an online survey was distributed to students from all medical schools in Australia through the General Practice Students Network.

**Main outcome measures:** Medical student perceptions on the effects of their future careers on the ability to maintain work-life balance and whether future parenting would impact their careers. Both quantitative and qualitative responses were collected.

**Results:**The majority of survey respondents believed their careers would have a moderate or significant impact on the ability to achieve work-life balance. Thematic analysis revealed medical students perceived medical careers as lacking flexibility, being time-consuming, and potentially detrimental to health. Surveyed students indicated both parenting goals and specialty choice needed to be considered when planning their career.

**Conclusions:** Australian medical students expressed significant concerns about their ability to juggle parenting and achieve work-life balance within the realities of a medical career.

## Introduction

Burnout is a well-described phenomenon with high prevalence amongst medical students and health professionals globally (Beyond Blue, 2013; Dahlin, Fjell and Runeson, 2010; Lemaire and Wallace, 2017). Burnout is a psychological syndrome of emotional exhaustion, depersonalization and reduced personal accomplishment that can lead to increased risk taking behaviours and depression ( Maslach and Jackson, 1981). Current and aspiring health professionals in Australia and worldwide have reported increased levels of burnout compared to the general population( Maslach and Jackson, 1981; Willcock *et al*., 2004). Physician burnout has many risk factors; these can often be identified or originate as early as medical school ( Krakowski, 1982). The Beyond Blue National Mental Health Survey of Doctors and Medical Students **(**2013) reported high rates of burnout and emotional exhaustion, particularly amongst females and young doctors (Beyond Blue, 2013).Notably, the most common stressor reported by doctors related to the need to balance work and personal responsibilities (26.8%). Inability to achieve work-life balance (WLB) is known to contribute to doctor burnout ( Rutherford and Oda, 2014).

The career aspirations of medical students potentially could be influenced by perceptions of future WLB ( Takahashi *et al.*, 2017). Females account for more than half of commencing medical student numbers in Australia, making balancing work and family commitments in medical careers an ongoing consideration ( Medical Deans of Australia and New Zealand Inc, 2020). There are disproportionate numbers of femalestraining in each specialty in Australia. Certain specialties are female-dominated, including obstetrics and gynaecology and general practice, whereas others have relatively lower proportions of female trainees, such as intensive care and surgery ( Medical Deans of Australia and New Zealand Inc, 2020). There is also evidence that medical graduates of both genders are concerned about the impact of vocational choice on family life and lifestyle ( Yen *et al.*, 2018; Alers *et al*., 2014).

There is a lack of research into medical students’ career aspirations with respect to perceived impact on WLB and parenting plans, particularly in Australia. In our study, data was analysed from a national cross-sectional survey of medical students in Australia regarding their career and lifestyle expectations. The aim was to explore the perceptions medical students have of their ability to achieve WLB after they graduate, and to assess perceptions of how parenting may influence their future career.

## Methods

The survey was developed in collaboration between General Practice Students Network (GPSN), General Practice Registrars Australia (GPRA), and the University of Wollongong. It aimed to understand Australian medical students’ awareness, knowledge and attitudes towards general practice.

Ethics approval; University of Wollongong (HREC number 2019/297).

### Participants and recruitment

An online survey was distributed across all medical schools in Australia through the General Practice Students Network. Any student currently enrolled in an Australian University Medical School was eligible to participate. Participation was voluntary and anonymous.

### Data Collection

Data was collected over eight weeks from September to October 2019. Quantitative and qualitative (free-text) responses were collected through the survey instrument.

### Statistical Analysis

Descriptive statistics were generated through Microsoft Excel **.** Thematic analysis of free-text responses was via constant comparison to identify significant phrases (by author S.L) and cross-checked (by author S.S). NVivo (v12.0, QSR International Pty. Ltd., Melbourne, Australia) was utilized for data management and grouped codes for thematic analysis.

## Results/Analysis

### Quantitative Results

A total of 1,129 medical students completed the survey (65.4% female). The respondents were mostly aged under 30 (90%), with 61.6% being 18-24 years old, and 29.6% being 25-30 years old ( Table 1). The majority of participants (95.1%) had no dependents and most (50.6%) listed their marital status as single.

**Table 1.** Demographic characteristics of survey respondents

	N	%
Total	(1,129)	100
**Gender**		
Female	738	65.4
Male	385	34.1
Prefer not to disclose	6	0.5
**Age**		
Under 18	1	0.1
18-24	695	61.6
25-30	334	29.6
31-40	82	7.3
41-50	15	1.3
50+	2	0.2
**# of Dependents**		
0	1074	95.1
1	17	1.5
2 +	34	3.0
Prefer not too disclose	4	0.4
**Marital Status**		
Single	571	50.6
Partner not living together	321	28.4
Defacto	127	11.2
Married	82	7.3
Other/prefer not to disclose	28	2.5

**Figure 1.**
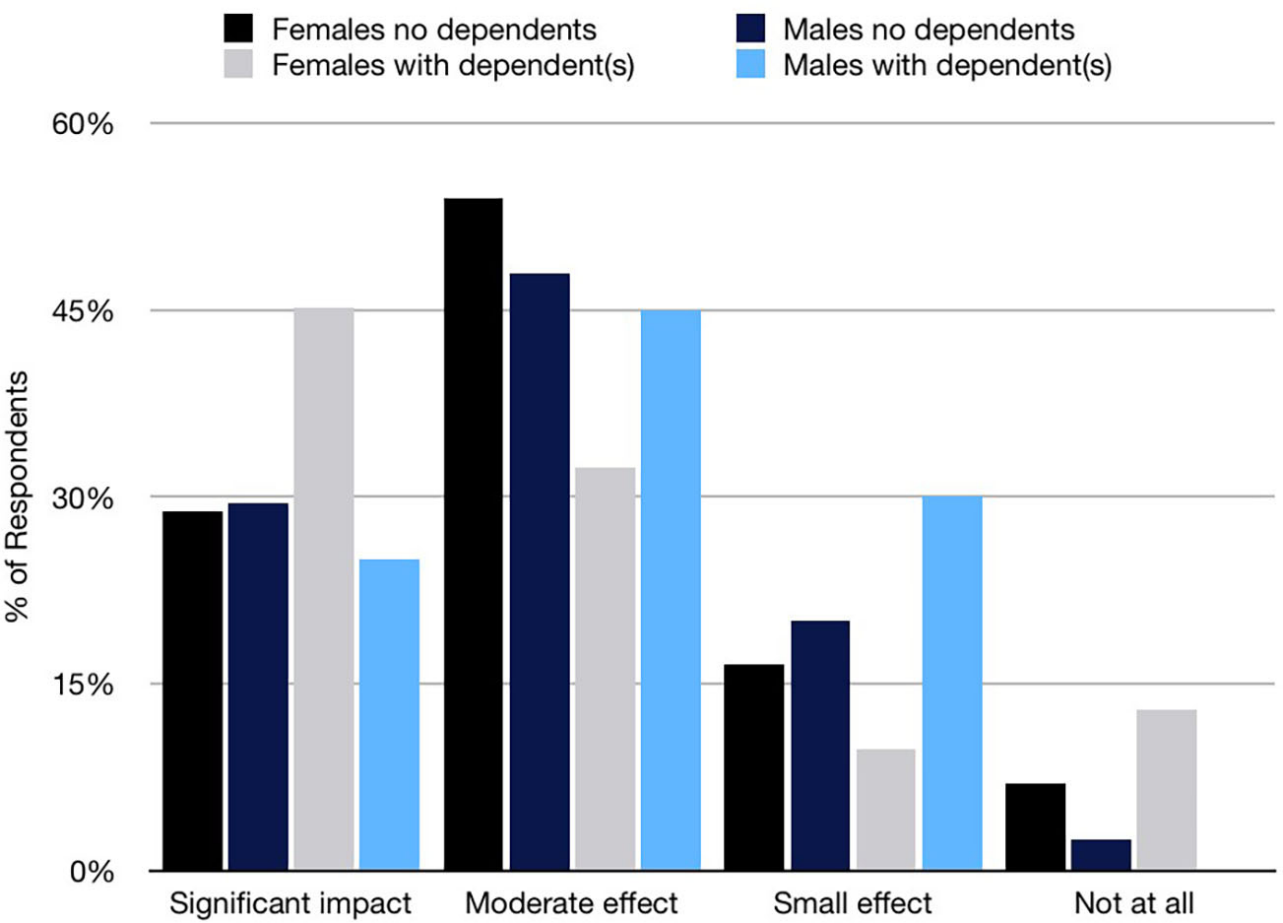
Medical students perceived difficulty in achieving WLB according to dependent status.

Students perceived that their future career pathway would cause difficulty in maintaining good WLB. The majority (80.8%) indicated their career would have a significant or moderate impact on WLB; however, a greater percentage of males (22.8%) compared to females (17.3%) believed their career would impact only a small amount or not at all. The findings were stratified based on respondents’ number of dependents and gender ( Figure 1). Most students with dependents (74.6%) indicated their career would significantly or moderately impact WLB. However, while 12.9% of women with dependents indicated that their career would not at all impact their ability to maintain good WLB, no men with dependents indicated the same.

The effect parenting would have on career pathway pursuit was identified in Table 2. Both females (47.7%) and males (43.9%) indicated that the impact would be moderate. Of those without dependents, a majority (47.2 %) also indicated a moderate impact. However, for those with dependents, most indicated that parenting would have a significant impact. Across all relationship categories, a minority (28.8%) viewed that parenting would have no or a small effect in career pursuit.

**Table 2.** Students’ perception of future parenting being a difficulty in pursuing career*

	Significant impact	Moderate effect	Small effect	No effect
**Gender**				
**Female (n=738)**	216 (29.3)	352 (47.7)	104 (14.1)	66 (8.9)
**Male (n=385)**	64 (16.6)	169 (43.9)	108 (28.1)	44 (11.4)
**Prefer not to disclose (n=6)**	2 (33.3)	0 (0)	3 (50.0)	1 (16.7)
**Dependents**				
**0 dependents (n=1074)**	265 (24.7)	507 (47.2)	198 (18.4)	104 (9.7)
**1 dependent (n=17)**	5 (29.4)	4 (23.5)	4 (23.5)	4 (23.5)
**2+ dependents (n=34)**	11 (32.4)	9 (26.5)	11 (32.4)	3 (8.8)
**Prefer not to disclose (n=4)**	1 (25.0)	1 (25.0)	2 (50.0)	0(0)
**Relationship Status**				
**Single (n=571)**	145 (25.4)	243 (42.6)	119 (20.8)	64 (11.2)
**Partner not living together (n=321)**	82 (25.5)	171 (53.3)	44 (13.7)	24 (7.5)
**Married or De facto (n=209)**	49 (23.4)	91 (43.5)	47 (22.5)	22 (10.5)

Subtitle: *data displayed as n(%) of total respondents to question: “To what extent do you think that parenting will be a difficulty for you in pursuing your career pathway following graduation from medical school?”

### Qualitative Results

Qualitative thematic analysis was performed across two free-text response questions asking why students believed parenting and WLB would be influenced by their medical career pursuit to varying degrees. The analysis revealed five major themes which are presented below (and Tables 3-7). Our survey identified the common perception that WLB is often compromised in a medical career and noted common perceptions of parenthood affecting their career plans.

#### Theme 1: The medical career is incredibly demanding of an individual’s time alongside other competing demands

The impact of the medical career on the availability of time was discussed ( Table 3).Students predicted a number of interests would be sacrificed due to lack of time and inflexibility of rosters. This was complicated by pressure to dedicate time towards further professional development, such as volunteering and research, to compete for increasingly limited specialty training positions. Students perceived that specialty training would be the most time-consuming, due to resume preparation and vigorous study. Most aspired to become consultants, who were believed to have the ideal WLB. Parenting was mentioned as a specific activity that would require significant time and compete with a medical career.

**Table 3.** Representative quotes for the theme “The medical career is incredibly demanding of an individual’s time alongside other competing demands”

Subtheme	Sample quote
***Sacrifice of external commitments***	• “To me, maintaining the relationships, interests and hobbies I have had for majority of my life before getting into medicine is very important... to give them up to solely focus on a career is giving up who I am as a person.” • “[I have] strong interests outside of medicine that I believe will suffer from long, unpredictable working hours”
***Pressure for further work***	• “I will find it hard to differentiate my time between the hospital, leisure and study for my future career” • “I’d always choose work first, at least until I get into the training program, then I can stop being so competitive.”
***Demand peaks during specific stages of training***	• “I think at certain times in my career (early on such as in internship and residency) I will be required to shift the balance more towards the work rather than life side”.

#### Theme 2: Family planning

Many students wanted a family but understood this to be considerably more difficult to balance with a medical career ( Table 4). Female students felt taking maternity leave would hinder their career progression. They also expressed concern over their “biological clock” coinciding with the duration of crucial training years, during which there was little flexibility. Students recognized parental leave policy as inadequately supportive of medical professionals having a family, regardless of career stage. Other students commented that they did not plan to have children, either from their own preferences or as a consequence of pursuing medicine. Students’ attitudes towards having children and maintaining desired WLB often depended on their spouse’s occupation. Those who had a spouse in a different occupation often identified that the spouse would take on more parental responsibility, while some with a spouse in the medical field suggested one partner would need to compromise their career.

**Table 4.** Representative quotes for the theme “Family planning”

Subtheme	Sample quote
***Choosing to have children***	• “I don’t want to take time off and be years behind my male counterparts in terms of career” • “It’s difficult as a woman to compete for specialties and remain relevant in your field whilst also wanting to be a dedicated mother.” “I don’t want to be a parent who doesn’t have time to raise their kids”
***Timing of parenthood***	• “My most fertile years will be during medical training” • “[There is a need to] time maternity leave appropriately so that it has minimal disruption to your training”
***Significance of spouse’s occupation for family***	• “My partner is also a medical student and if we were to remain together I anticipate my career will likely become second priority... he is one year ahead of me and likely will always be ahead of me in his training.” • “I have always assumed that I cannot be the primary caretaking parent because of my choice to undertake a medical career”

#### Theme 3: Potential impact of career on mental and physical health

Many students were aware that their future career could affect their mental and physical health ( Table 5). Some noted the time-pressures of a medical career would permit less time for self-care, including activities such as exercising, cooking healthy meals and socialising. Others were concerned about the emotional burden of medicine. Some mentioned the difficulty of leaving work behind, as their actions have direct effects on patients. This was thought to improve with shift work, as the responsibility could be shared and they would not feel they were “abandoning the patient”.

**Table 5.** Representative quotes for the theme “Potential impact of career on mental and physical health”

Subtheme	Sample quote
***Reduced time for self-care***	• “There is an expectation in medicine to give up your lifestyle outside of work”
***Stress and mental health***	• “Long hours, and the difficulty of this profession of leaving work at work will definitely affect work/life balance”

#### Theme 4: Perceived lack of flexibility in the career

Student stated that the structure of medical careers did not facilitate healthy WLB ( Table 6). Students perceived that this would be worse in certain specialties that demanded frequent on-call shifts. These were perceived to be both physiologically stressful and a source of pressure in meeting other commitments. Students also described an uncertainty about future relocation for employment and training which were thought to be outside of their control. The removal of familiar support networks in these situations was stated as a barrier to achieving good WLB.

**Table 6.** Representative quotes for the theme “Perceived lack of flexibility in the career”

Subtheme	Sample quote
***Irregular work hours***	• “[I am] concerned about evening ward covers and weekend shifts, being overloaded with admin tasks that require me to either arrive early for my shift, work through my lunch break, or stay late” • “Many people have told me of missing family events such as birthdays. Furthermore, one of the surgical consultants asked for someone to fill in for him as he had not seen his wife in 4 days and it was their anniversary coming up”
***Future relocation***	• “I will find it hard to differentiate my time between the hospital, leisure and study for my future career” • “I’d always choose work first, at least until I get into the training program, then I can stop being so competitive.”

#### Theme 5: Choice of future specialty

Some students indicated they had already chosen their future specialty, and this was influenced by their existing values regarding WLB and family ( Table 7). Some students with dependents felt that their specialty preference was solely dictated by their parental responsibilities. These students tended to opt for specialties perceived to have a better WLB such as general practice, psychiatry and pathology. Other students felt prepared to sacrifice their WLB and sometimes parenthood in order to pursue their career passions, which they felt would increase their overall level of personal satisfaction.

**Table 7.** Representative quotes for the theme “Choice of future specialty”

Subtheme	Sample quote
***Specialty preferences restricted to those with a better WLB***	• “I am currently pregnant and my speciality choice will have to accommodate.” • “Being slightly older and married, I don’t necessarily feel like I will like to strive towards a speciality that may take me away from my growing family.” • “Personally, parenting will be of a higher priority than furthering my career as I hold a strong family-centric ethos. This will limit my career paths”
***WLB sacrificed for passion***	• “It’s not that it’s difficult or not. It’s what I am willing to give up” • “The pathway I’d like to go into doesn’t have the best work-life balance however I don’t think I’d be happy doing anything else”

### Discussion

This study is the first to examine the perceptions of Australian medical students on how their future medical careers would affect WLB and parenthood. Quantitative survey data indicated a majority of students believed their career would have a significant or moderate impact on WLB. Similarly, most viewed future parenting as likely to cause a moderate or significant impact on their career. Qualitative analysis revealed medical students perceived medical careers as lacking flexibility, being time-consuming, and potentially detrimental to their health. Many students believed lifestyle, parental plans and specialty choice needed to be considered when planning a medical career in order to achieve their ideal WLB.

Views expressed by our survey respondents are consistent with international literature, in that good WLB is difficult to achieve across the medical specialties ( Rich *et al.*, 2016; Shrestha and Joyce, 2011). One common theme that arose was the notion of long hours worked as a trainee, particularly during crucial times in junior years. While it is known that long working hours is a risk factor for mental and physical ill-health in the general population( Afonso, Fonseca and Pires, 2017), there is a common belief that these hours are required for learning in a junior doctor setting ( Petrie *et al.*, 2020). However, Petrie *et al.* showed a link between long work hours and significantly higher likelihood of mental disorders and suicidal ideation among junior doctors ( Petrie *et al.*, 2020). In other countries there have been moves to limit the number of hours worked by trainees, whereas in Australia working hours are defined by employment agreements that differ according to state ( O’Grady *et al.*, 2012). The realities of long work hours and the conditions in Australia may explain the perceptions of the medical students surveyed. The lack of flexibility in medical careers, such as irregularity of hours and lack of control of placement locations was also expressed as a barrier to achieving good WLB. This is consistent with recent survey results of Australasian surgical trainees which found desire for part-time work options greatly outweighed availability ( Mcdonald *et al*., 2013). Thus, most medical students surveyed expressed negative expectations of their ability to achieve good WLB in their future careers.

Medical students surveyed considered their WLB goals when selecting a future specialty. Many students believed certain specialties, such as general practice, psychiatry and pathology, would enable a better WLB. While it is a common perception that general practice is a ‘lifestyle-friendly’ specialty, research has shown that only half of the general practice workforce in Australia report good WLB with high levels of burnout within the profession ( Shrestha and Joyce, 2011). Thus, it is possible that medical students’ perceptions may underestimate the demands of general practice. Various opinions were given about specialties involving shift work, such as emergency medicine and intensive care. While some respondents mentioned negative aspects of shift work, many perceived shift work to be beneficial to WLB, allowing flexibility and opportunities to spend time with family and friends. This contrasts with the published research documenting negative impacts of shift work on both WLB and physical and mental health ( Hermansson *et al*., 2018; Ahmed-Little, 2007). Shift work has been linked to a range of medical conditions from obesity to cardiovascular disease ( Liu *et al.*, 2018). The incongruence between the documented negative effects of shift work with the views of survey respondents may be explained by the lack of exposure medical students have to the realities of shift work.

A majority of surveyed students considered future parenting as likely to impact their career. This is in agreement with existing literature documenting increased family strain for general surgical residents with children than those without ( Sullivan *et al.*, 2012). Both male and female medical students in our survey were equally likely to view parenting as significantly affecting their future careers, in contrast to a similar survey conducted in Japan. Takahashi *et al*. found that Japanese female students expected a higher childcare and housework burden, and a greater percentage would consider working part-time or temporarily leaving work after they had children, compared to male students ( Takahashi *et al.*, 2017). This may reflect cultural and workforce factors, as Japan has exceptionally low proportions of women who remain in the workplace after having children, and only 20% of physicians are female ( Brinton and Oh, 2019; Matsui *et al.*, 2019).

The main strengths of our study were the large-scale nature of the cohort survey and the representation from all medical schools across Australia. Moreover, the methodological approach allowed both quantitative analysis and exploration of opinions regarding WLB and parenting in medical careers. Nonetheless, the self-selected nature of participants may mean that the views are not reflective of the general medical student population. In addition, the majority of the students surveyed were single with no dependents. Increased representation from students married or in de facto relationships and/or those with dependents may have provided different perspectives.

### Conclusion

Our survey revealed that Australian medical students perceived there would be challenges associated with balancing life and pursuing a medical career. Many medical students considered parenting in planning their future careers. These results indicate a need for ongoing research into factors contributing to poor WLB in the medical profession, and an increased clinical exposure of medical students to shift work in their clinical years. The development of part-time vocational medical training and evaluation of the policies and programs for health systems may facilitate flexibility in working conditions particularly for junior doctors in the future.

### Take Home Messages

• There is increasing numbers of junior doctors experiencing burnout and suicide, medical students perceive their work-life balance to be very difficult.
• Female medical students are graduating medical school in increasing numbers and developing experiences to improve perceptions of careers that may lead to a good work-life balance, and particularly, balancing parenting and career is desired.

### Notes On Contributors

**Dr Sarika Suresh,** is a junior doctor working at Concord Repatriation General Hospital in Sydney. She graduated MD with Distinction from the University of Melbourne in 2019 and is currently completing a Master of Medicine (Clinical Epidemiology) at the University of Sydney. (ORCID iD: https://orcid.org/0000-0003-1525-6749.)

**Dr Rebekah Hoffman**, MBBS, BSci, MPH, MSurg, MSpMed, GDAAD, DCH, GAICD, FRACGP is a GP in Sydney and an Academic / Senior Lecturer at the University of Wollongong. She has interests across the wellbeing and career balance of medical students and junior doctors.

**Sue Liu,** is a medical student studying at Monash University, Australia.

**Dr Andrew Gosbell**, PhD, PhD, GAICD, FIML is the Chief Executive Officer of General Practice Registrars Australia. He has a background in laboratory sciences and broad research interests including medical education, health workforce and public health with translational experience in advocacy and governance.
