# Supplementary material for: Australian medical student expectations of work-life balance as a doctor
Source: MedEdPublish (2016). 2020 Nov 17;9:256. [Version 1] doi: 10.15694/mep.2020.000256.1 (PMC10697482; doi:10.15694/mep.2020.000256.1)
Supplement: Peer review history

# Reviewer report: Reviewer response for version 1

**DOI:** 10.21956/mep.20154.r31135

**Published:** 2021-07-18

**Authors:** Helena Filipe

**Affiliations:** 1 Hospital of the Armed Forces/PL-EMGFA

This review has been migrated. The reviewer awarded 4 stars out of 5 I read with interest this paper on work-life balance considering physicians’ ancient highly demanding professional committal within the contemporary rapidly changing work environments and societal increasing expectations. Bringing this theme to the medical schools’ benches is a relevant piece in the significant need of approaching medical professionals’ wellbeing and burnout management with a holistic perspective. Kudos for conducting this important, needs assessment investigation.It is interesting to see how this can shape the future of medical specialties and professionals leaving behind a field of inquiry as how the other least chosen will be left and the related healthcare will evolve.A word of remark for the sample dimension, highlighting the high female predominance and the mixed-methods approach to extract deeper conclusion knowledge from the investigation.Thank you for the research translated to the paper that highlights a core theme in the medical profession and makes us reflect about the profession and the mission of being a physician.I would like to encourage further study on amplifying the sample to other regions of the world and reflecting on how people in healthcare leadership positions could meaningfully act to revert “poor WLB in the medical profession”.

Reviewer Expertise:

NA

No decision status is available

# Reviewer report: Reviewer response for version 1

**DOI:** 10.21956/mep.20154.r31136

**Published:** 2021-02-23

**Authors:** Richard Hays

**Affiliations:** 1 James Cook University

This review has been migrated. The reviewer awarded 3 stars out of 5 This paper documents an issue that is highly topical, not just in Australia. Medical educators are seeing more learners (both undergraduate and postgraduate) with anxiety, sometimes associated with concern that 'achieving work life balance' will be difficult. I welcome the documentation of this with a survey of learners and the qualitative analysis seems to produce results in line with experience. There are however some conceptual and methodological issues that, if addressed, would make research like this more meaningful. The first is that the response rate is probably low - about 30% - making interpretation of any quantitative data more difficult. A response rate should be listed and acknowledged as a limitation. Selection bias is possible. The second is that the term 'work-life balance' needs clearer definition. Is this a personal, subjective 'sense' that varies between people and across different stages of life? Some exploration of this would be interesting. The third is that a focus on addressing the problems may be more valuable than a description of them. From my perspective it is interesting that concerns about work-life balance may not have changed much for decades, despite much shorter working hours, improved support in the workplace and increasing availability of part-time specialty training. My fourth concern is that the assertion in the 'Take home message's that suicide and burnout rates are increasing does not come from the data reported. Is this correct, or are we just hearing and talking about it more - more awareness may be a good thing, but (sadly) this has been happening for a long time, yet rarely reported or discussed Back to the study, a solution-focus may be more useful. What could we (collectively) do to make careers challenging, rewarding and less impactful on personal lives? All ideas are welcome! Addressing these issues would merit an extra star.

Reviewer Expertise:

NA

No decision status is available
